# Value of early pregnancy ultrasound combined with ultrasound score in the evaluation of placenta accreta in scar uterus: A retrospective cohort study

**DOI:** 10.1097/MD.0000000000037531

**Published:** 2024-03-15

**Authors:** Cuigai Wang, Zhiyuan Wang

**Affiliations:** a Department of Ultrasonography, Hebei reproductive maternity hospital, Shijiazhuang City, Hebei Province, China; b Department of Ultrasound, Zhengding County People’s Hospital, Shijiazhuang City, Hebei Province, China.

**Keywords:** early pregnancy ultrasound, placenta accreta, scar uterus, ultrasound score

## Abstract

The objective of this study is to investigate the value of early pregnancy ultrasound combined with ultrasound score (USS) for the evaluation of placenta accreta (PA) in scar uteri. Thirty cases of PA in scar uteri diagnosed by ultrasound at our hospital between June 2021 and June 2022 were selected retrospectively (observation group). In addition, 30 patients had placenta attached to the anterior wall of the uterus and covered the internal orifice of the cervix; however, no PA was selected in the same period (control group). The results of surgical pathology and ultrasound examination in the first trimester of pregnancy (11–14 weeks of pregnancy, fetal top hip length 4.5–8.4 cm) were analyzed. Ultrasonic image characteristics of the 2 groups were scored using an ultrasonic scoring scale. The ultrasonic signs and ultrasonic scores of the 2 groups were analyzed. The diagnostic value of ultrasound and USS for PA in the scarred uterus alone and in combination was analyzed based on the gold standard of surgical and pathological results. The rich blood flow signal at the junction of the uterine serosa and bladder, the rate of blood flow in the cavity of the placental parenchyma, the thinning rate of the myometrium after placenta, and the abnormal rate of the low echo area after placenta in the observation group were significantly higher than those in the control group (*P* < .05). The USS of the observation group was significantly higher than that of the control group (*P* < .05). The sensitivity (93.33%) and accuracy (95.00%) of the combined examinations were significantly higher than those of ultrasound (70.00% and 83.33%, respectively) (*P* < .05). The sensitivity and accuracy of combined examination were slightly higher than those of USS examination (83.33% and 90.00%), but the difference was not statistically significant (*P* > .05). There was no significant difference between the specificity of combined examination (93.33%) and ultrasound (96.67%) and USS (96.67%) (*P* > .05). Early pregnancy ultrasound and USS evaluation have high application value in the diagnosis and evaluation of early scar uterine PA. The combination of the 2 methods can further improve the sensitivity and accuracy of diagnosis.

## 1. Introduction

Placenta accreta (PA) is the reduction or loss of uterine decidua caused by primary hypoplasia of the uterine decidua, traumatic endometrial defect, infection, etc.^[1]^ The physiological crack line of the spongy layer between the placenta and uterine decidua disappears, resulting in close adhesion of the maternal leaves of the placenta to the basal layer of the decidua/invasion of the myometrium.^[1,2]^ Studies have pointed out that the current common clinical PA is mainly caused by traumatic endometrial defects, and a history of cesarean section (CS) is the main risk factor.^[3]^ Research shows that after CS, the growth of the endometrium around the incision is poor, the muscle layer is weak, the scar is gradually replaced by fibrous tissue, and its elasticity is reduced.^[4]^ The implantation of the gestational sac at the time of second pregnancy can cause poor formation of the decidua at the bottom; the villi penetrate the myometrium and cause PA.^[4–6]^

In recent years, with the increasing rate of CS, the incidence of PA in scar uteri continues to rise, which has great harm.^[7]^ Bleeding, perforation, rupture, and other adverse events may occur at the beginning of the pregnancy.^[8]^ If patients do not receive timely and effective intervention, they will pose a huge threat to their life and health.^[7–9]^ Therefore, early accurate diagnosis of PA in scarred uteri is of great significance. Ultrasound is a commonly used diagnostic technique in obstetrics and gynecology, and has the advantages of strong repeatability, simple operation, low cost, and noninvasiveness, and can be used as the preferred examination method for PA.^[10]^ However, examination results are easily affected by doctors’ experience, examination time, placental attachment position, PA depth, etc.^[10,11]^ Some studies have used a quantitative ultrasound scoring scale for prenatal diagnosis, which can more accurately distinguish the different degrees of implantation.^[12]^ The quantitative scoring scale comprehensively considered the influence of placenta previa type, 2-dimensional ultrasound, and color Doppler ultrasound on a variety of typical abnormal signs, as well as focusing on the most difficult ultrasound features of bladder and cervical invasion during surgery; the degree of implantation was evaluated according to the score; compared with simple subjective qualitative diagnosis, it is more comprehensive and standardized, and it excludes different ultrasonic signs selected by different ultrasound physicians according to personal experience, and gives the possibility of implantation judgment of different importance; the rate of missed diagnosis and misdiagnosis was reduced.^[11,12]^ There are few similar reports about the safety and effectiveness of early pregnancy ultrasound combined with ultrasound score (USS) tables in predicting PA in scar uteri in China.

Based on this, this study retrospectively analyzed the ultrasound examination results and ultrasound scale scores of patients with scarred uterus PA in our hospital. We aimed to explore the value of early pregnancy ultrasound combined with USS in the diagnosis and evaluation of disease.

## 2. Materials and methods

### 2.1. Methods

A total of 30 patients with PA in a scarred uterus diagnosed by ultrasound in our hospital from June 2021 to June 2022 were selected as the observation group, and 30 patients whose routine prenatal ultrasound examination showed that the placenta was attached to the anterior wall of the uterus and covered the internal orifice of the cervix, but no placenta accrete was found, were selected as the control group.

All procedures performed in the study involving human participants were in accordance with the ethical standards of the institutional and/or national research committee(s) and the Helsinki Declaration (as revised in 2013). The requirement for informed consent was waived by the ethics committee due to the observational and retrospective nature of the study.

#### 2.1.1. Inclusion criteria.

- During the regular prenatal examination in our hospital, ultrasound diagnosis of PA was made in early pregnancy.
- The gestational age was 11 to 14 weeks.
- Have a history of CS.
- The fetal top hip length was 4.5 to 8.4 cm.
- The image data are complete.

#### 2.1.2. Exclusion criteria.

- The ultrasound image quality is poor, and the lower segment of the anterior wall of the placenta and uterus cannot be diagnosed and displayed.
- Patients who did not undergo pathological examination or failed to track the pregnancy outcome.
- Patients with mental disorders.
- Abnormal placental morphology.
- Multiple pregnancy.
- Patients with benign and malignant tumors.
- Patients with hematological diseases or abnormal coagulation function.

### 2.2. Ultrasonic examination

The equipment is iu22 color Doppler ultrasound diagnostic instrument of Philips company in the United States. The frequency of ultrasonic probe was set to 3.5 MHz, and the patient was informed to fill the bladder before examination. Assist them to take the supine position and effectively expose the pelvic cavity and abdomen. First, the fetus (including placental attachment position, amniotic fluid depth, top hip length, etc) and its appendages were routinely examined; the relative position between the edge of the placenta and the internal orifice of the cervix was examined; continuously adjust the direction of ultrasonic probe to clarify the relationship between the edge of placenta and the internal orifice of cervix uteri; the presence of PA was determined by referring to the placenta, posterior placental space, peripheral blood flow (BF) status, echo in placental parenchyma, myometrial integrity of anterior uterine wall, BF signal, and echo at uterine serosa bladder junction; and record its internal echo, shape, size, and other information in detail; reexamination was carried out for suspected PA patients.

### 2.3. Diagnostic criteria

For the ultrasonic diagnosis of PA, refer to the diagnostic criteria of the guidelines for the diagnosis and treatment of PA (2015): the normal structure of the placental site is disordered, the normal hypoechoic area behind the placenta disappears or becomes thinner, the blood vessels at the junction of the uterine serosa and bladder are rich, and there is focal or diffuse intraluminal BF in the placental parenchyma.^[13]^ For pathological diagnosis of placenta accrete, it was referred to the study by Zhang et al.^[14]^

### 2.4. USS

The USS of scarred uterus PA was established according to the results of ultrasound examination, and a total score of ≥3 was used as the judgment standard for PA (Table 1).^[12]^

**Table 1:** Ultrasonic scoring scale of scar uterus placenta implantation.

Ultrasonic signs	Classification	Ultrasonic score (score)
Partial or complete disappearance of posterior placental space	No	0
	Yes	2
Thickness of the thinnest uterine myometrium at placental attachment	>2 mm	0
	1–2 mm	1
	<1 mm	2
Extensive or focal intraplacental BF	None	0
	Visible	1
BF of uterine serosa bladder interface at placental attachment	None or rare	0
	Richness	2
	Rich and disorganized	3

BF = blood flow.

### 2.5. Observation index

(1) Characteristics of ultrasonic signs. (2) USS. (3) The value of ultrasound and USS in the diagnosis of PA in scarred uterus alone and in combination.

### 2.6. Statistical methods

All data were analyzed using the SPSS software (version 25.0; IBM Corp, Armonk, NY). The measurement data are expressed as mean ± standard deviation, and an independent sample *t* test was used for comparison between groups. The counting data are represented by the number of use cases using the chi-square test. When *P* < .05, the differences were considered statistically significant.

## 3. Results

**Figure 1.**
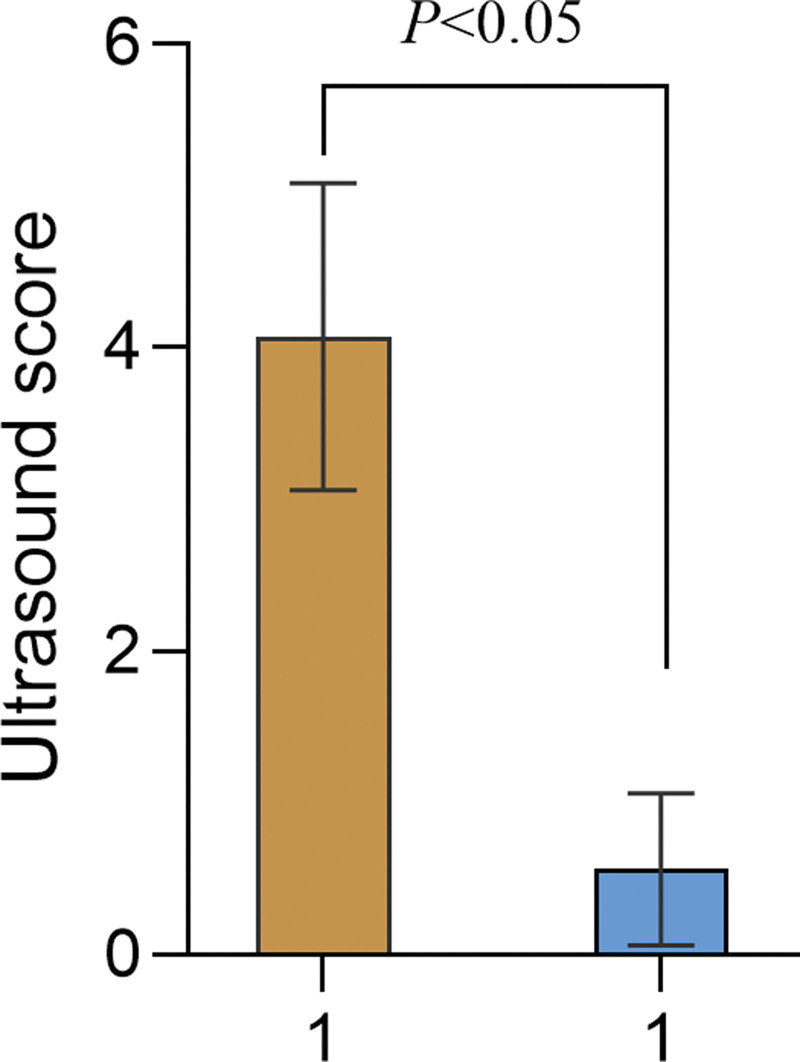
Comparison of USSs between the 2 groups. USS = ultrasound score.

There was no significant difference in age, body mass index, gravidity, and number of CS between the 2 groups (*P* > .05), but the hysterectomy rate in the observation group was higher than that in the control group (Table 2). The rich BF signal at the junction of the uterine serosa and bladder, the rate of BF in the cavity of the placental parenchyma, the thinning rate of the myometrium after placenta, and the abnormal rate of the low echo area after placenta in the observation group were higher than those in the control group (*P* < .05) (Table 3). The USS of the observation group was significantly higher than that of the control group (*P* < .05) (Fig. 1). Considering the surgical and pathological results as the gold standard, 21 cases of PA in the scar uterus were detected in the observation group and 29 cases of PA in the non-scar uterus were detected in the control group; 25 cases of PA in the scar uterus were detected in the observation group and 29 cases of PA in the non-scar uterus were detected in the control group; 29 cases of PA in the scar uterus were detected in the observation group and 28 cases of PA in the non-scar uterus were detected in the control group (Table 4). The sensitivity (93.33%) and accuracy (95.00%) of combined examination were significantly higher than those of ultrasound (70.00%, 83.33%) (*P* < .05), and the sensitivity and accuracy of combined examination were slightly higher than those of USS examination (83.33%, 90.00%), but the difference was not statistically significant (*P* > .05). However, there was no significant difference between the specificity of combined examination (93.33%) and ultrasound examination (96.67%) and USS examination (96.67%) (*P* > .05) (Table 5).

**Table 2:** Comparison of baseline data between 2 groups.

Group	Age (yr)	BMI (kg/m²)	Gravidity	Number of CS	Hysterectomy (n)
Observation group (n = 30)	31.77 ± 2.78	25.62 ± 3.13	2.37 ± 0.56	1.97 ± 0.72	8 (26.67)
Control group (n = 30)	32.23 ± 3.81	24.96 ± 2.82	2.50 ± 0.63	1.87 ± 0.78	2 (6.67)
*t*/*χ*^2^	−0.542	0.853	−0.869	0.518	4.320
*P*	.590	.397	.388	.606	.038

BMI = body mass index, CS = cesarean section.

**Table 3:** Comparison of ultrasonic signs between the 2 groups.

Group	n	The abundance rate of BF signals at the junction of uterine serosa and bladder	Rate of BF in the cavity of placental parenchyma	Retroplacental myometrium thinning rate	Abnormal rate of low echo area after placenta
Observation group	30	16 (53.33)	16 (53.33)	9 (30.00)	30 (100.00)
Control group	30	0 (0.00)	0 (0.00)	0 (0.00)	1 (3.33)
*χ* ^2^		21.818	21.818	8.366	56.129
*P*		<.001	<.001	.004	<.001

BF = blood flow.

**Table 4:** Detection of ultrasound and USS for PA in scar uterus

Ultrasound	Pathological results		Total	Ultrasound score	Pathological results		Total	Combined examination	Pathological results		Total
	+	−			+	−			+	−	
+	21	1	22	+	25	1	26	+	29	2	31
−	9	29	38	−	5	29	34	−	1	28	29
Total	30	30	60	Total	30	30	60	Total	30	30	60

PA = placenta accreta, USS = ultrasound score.

**Table 5:** Comparison of ultrasound and USS in the diagnosis of PA in scar uterus.

Diagnosis mode	Sensitivity	Specificity	Accuracy
Ultrasound	70.00% (21/30)	96.67% (29/30)	83.33% (50/60)
USS	83.33% (25/30)	96.67% (29/30)	90.00% (54/60)
Combined examination	93.33% (28/30)	93.33% (28/30)	95.00% (57/60)
*χ*^2^/*P* (ultrasound vs combined examination)	5.455/.020	0.000/1.000	4.227/.040
*χ*^2^/*P* (ultrasound score vs combined examination)	0.647/.421	0.000/1.000	1.081/.298

PA = placenta accreta, USS = ultrasound score.

## 4. Discussion

The results of this study show that early pregnancy ultrasound and USS evaluation have a certain application value in the diagnosis and evaluation of early scar uterus PA. Lu et al^[13]^ pointed out that ultrasound is an important inspection measure for placenta previa with PA, and the combination of transabdominal ultrasound and transvaginal ultrasound is used for the comprehensive diagnosis of placenta previa with PA, with a diagnostic accuracy of 91.89%. The diagnostic rate of PA was 45.95%, confirming that the combination of the two can improve the accuracy of disease diagnosis.^[15]^ At the same time, ultrasonic examination is not only simple to operate, convenient to apply, and highly repeatable but can also effectively improve the image definition by improving the resolution of the probe. The ultrasonic beam can enter through the back of the bladder and the back of the lateral abdomen to prevent infection by reverberation artifacts and effectively present the lesions.^[10]^ Zhao et al^[16]^ showed that the rate of placenta previa, the rate of placenta vortex, the disappearance rate of posterior placental space, the thinning or disappearance rate of posterior placental muscle layer, and the rich rate of posterior placental BF in patients with PA were significantly higher than in those without PA. This shows that ultrasound examination can determine whether PA occurs in patients using relevant imaging features, which is consistent with the findings of this study. However, conventional ultrasound examination is easily affected by subjective factors and clinical experience, and the ultrasound scoring system can compensate for its shortcomings^[12,13]^ Chen et al^[17]^ explored the evaluation value of the ultrasound scoring system in the treatment of PA disease; according to different USSs, patients with PA were divided into low, medium, and high groups, and the results showed that the risk of bleeding increased with an increase in USS, indicating that the ultrasound scoring system has high application value in the diagnosis and treatment of PA. Zhang et al^[14]^ discussed the application value of prenatal placental USS in PA in the second and third trimesters of pregnancy, and the results showed that the USSs of patients with PA were significantly higher than those of patients without PA; the negative predictive value, positive predictive value, specificity, sensitivity, and accuracy of disease diagnosis can be significantly improved by combining MRI with USS. The results of this study also showed that the sensitivity and accuracy of combined diagnosis were significantly higher than those of conventional ultrasound examination and slightly higher than the evaluation of USS, but there was no significant difference between the two groups. It was confirmed that the diagnostic value of the ultrasound scoring system was higher than that of conventional ultrasound examination, and the combination of the 2 can effectively improve the sensitivity and accuracy of disease diagnosis, reduce the risk of missed diagnosis and misdiagnosis, and ensure that patients receive targeted intervention as soon as possible.^[18]^ Ye et al^[19]^ showed that the ultrasound scoring system is a comprehensive system of typical abnormal signs of disease, which is scored according to its severity, and the degree of implantation is evaluated by different scores; compared with the subjective qualitative diagnosis of conventional ultrasound, it is more standardized and comprehensive, and can avoid the shortage that the operator chooses different ultrasonic signs and endows different importance with implantation judgment due to personal operation technology and clinical experience, which can effectively reduce misdiagnosis and missed diagnosis rates. The ultrasound scoring system can predict the type and degree of implantation to guide patients in terminating pregnancy and effectively make preoperative preparations. Such as multidisciplinary collaborative management, reasonable selection of surgical methods, etc.^[19,20]^ At the same time, Yang et al^[21]^ pointed out that ultrasound examination can determine the BF status of the cavity BF in the placental parenchyma and the junction of uterine serosa and bladder; the ultrasound scoring system can confirm whether the subject has PA at the thinnest part of the uterine myometrium with the help of conventional ultrasound signs and other information. The combination of the two can realize complementary advantages and disadvantages and effectively improve the value of disease diagnosis and treatment evaluation. Based on this, it is believed that in clinical practice, to avoid missed diagnosis or misdiagnosis to the greatest extent, conventional ultrasound and ultrasound scoring systems can be integrated to carry out inspection and evaluation for patients with early scar uterine PA.^[19–21]^

### 4.1. Limitations

First, this was a single-center retrospective study. Incomplete medical records and the bias in recalling medical history increase the complexity of the study and may be prone to selection bias. Second, the two groups were not randomly assigned, and the baseline information may be unbalanced and biased, which is also a limitation of our retrospective study. Third, the ultrasonic signs may be affected by human or technical factors. Fourth, the sample size was small, the degree of PA was not analyzed, and the pregnancy and neonatal outcomes of the patients were not analyzed. Finally, we will continue to conduct higher-quality research in the future to verify the content of this conclusion.

## 5. Conclusion

Early pregnancy ultrasound and USS evaluation have high application value in the diagnosis and evaluation of early scar uterine PA. The combination of the two methods can further improve the sensitivity and accuracy of diagnosis.

## Author contributions

**Conceptualization:** Cuigai Wang.

**Data curation:** Cuigai Wang.

**Formal analysis:** Cuigai Wang.

**Investigation:** Zhiyuan Wang.

**Writing – original draft:** Cuigai Wang.

**Writing – review & editing:** Cuigai Wang.
